# Updates and controversies in contemporary grading of clear cell renal cell carcinoma and papillary renal cell carcinoma

**DOI:** 10.1111/his.70177

**Published:** 2026-05-29

**Authors:** Gladell P. Paner, Mahul. B. Amin, Jung Woo Kwon, Liang Cheng, Holger Moch

**Affiliations:** ^1^ Department of Pathology and Surgery (Urology) University of Chicago Chicago Illinois USA; ^2^ Department of Pathology and Laboratory Medicine University of Tennessee Health Science System Memphis Tennessee USA; ^3^ Department of Urology USC Keck School of Medicine Los Angeles California USA; ^4^ Department of Pathology and Laboratory Medicine and Surgery (Urology), Brown University Warren Alpert Medical School, Legorreta Cancer Center Brown University Health Providence Rhode Island USA; ^5^ Department of Pathology and Molecular Pathology University Hospital Zurich Zurich Switzerland

**Keywords:** carcinoma, grade, ISUP, prognosis, renal, WHO

## Abstract

A decade into the formal adoption of the World Health Organization (WHO)/International Society of Urological Pathology (ISUP) grading system for clear cell renal cell carcinoma (CCRCC) and papillary renal cell carcinoma (PRCC), newer grading concepts, innovative approaches and some issues have emerged. A comprehensive review of the literature on the WHO/ISUP grading system and other grading approaches for CCRCC and PRCC was conducted. Updates and issues are presented on the following: (1) validation studies on WHO/ISUP grading; (2) grade elements including heterogeneity, grades 1 versus 2, types of multinucleated tumour cells, ‘grade spectrum’ of spindle cells, impact of sarcomatoid change in grade 4 RCC, percent (%) high grade, % sarcomatoid change and rhabdoid change versus sarcomatoid change; (3) grade applications including observer agreement, needle biopsy grade accuracy (correlation with nephrectomy grade) and grading of multifocal RCCs; (4) grade in papillary tumours including heterogeneity and on small (≤1.5 cm) neoplasms; and (5) novel grading or risk categories including incorporation of necrosis into grading and pattern or architectural‐based grading. Practice guidance for some of the issues is provided when feasible or where data are sufficient. This review highlights the recent updates and controversies in the use of WHO/ISUP grading and on novel approaches to grading for CCRCC and PRCC. This review may serve as a best practice guide in addressing some of the grading issues and will help identify gaps in our understanding and use of grading for CCRCC and PRCC that can inform future research.

## Graphical abstract

New concepts, innovations and some issues have emerged since the adoption a decade ago of WHO/ISUP grading for CCRCC and PRCC. Continued use of the WHO/ISUP grading for CCRCC and PRCC has been upheld by newer studies, and practice guidance for some of the grading issues, where data are available, is provided.

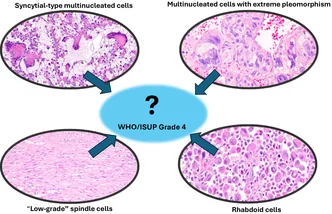

**Abbreviations**
CAP: College of American Pathologists
CCRCC: Clear cell renal cell carcinoma
ChRCC: Chromophobe renal cell carcinoma
CSS: Cancer‐specific survival
DFS: Disease‐free survival
EMT: Epithelial‐mesenchymal transition
ICCR: International Collaboration on Cancer Reporting
ISUP: International Society of Urological Pathology
MCDA: Multinucleated cell with degenerative atypia
MFS: Metastasis‐free survival
NB: Needle biopsy
OS: Overall survival
PFS: Progression‐free survival
PRCC: Papillary renal cell carcinoma
RCC: Renal cell carcinoma
RFS: Recurrence‐free survival
STMC: Syncytial‐type multineucleated cell
WHO: World Health Organization

## Introduction

Grade is a crucial pathological factor in the management and prognostication of renal cell carcinoma (RCC).^1^, ^2^ For >70 years, the manner and applicability of grading for RCC have considerably evolved largely due to adjustments in its components and to later adaptations to the growing number of RCC subtypes.^3^, ^4^ Only in the prior decade has the World Health Organization (WHO)/International Society of Urological Pathology (ISUP) grading system been formally adopted for clear cell RCC (CCRCC) and papillary RCC (PRCC).^5^, ^6^ The WHO/ISUP grade has now been integrated into major urological guidelines and risk stratifications for CCRCC and PRCC,^1^, ^7^ and is considered a core element when reporting RCC in renal biopsy (RB) and nephrectomy specimens by the International Collaboration on Cancer Reporting (ICCR) and the College of American Pathologists (CAP).^8^, ^9^

The grading of other RCC subtypes beyond CCRCC and PRCC, including the use of the WHO/ISUP grading system, is still under investigation. Recently, three authors (G.P.P., M.B.A. and H.M.) provided updates on grading the less common RCC subtypes that highlighted the gaps in grading and offered guidance for surgical pathologists on grading considerations for these tumours.^4^ This practice guidance has also been incorporated into the 2022 WHO blue book.^10^ This review will focus on CCRCC and PRCC, where the use of WHO/ISUP grading is established, and will cover updates and issues in the application of grading and the grade components. Further, innovative approaches including incorporation of other elements (e.g. necrosis, architecture) in grading will be explored. We also hope that this review will shed light on the gaps in our understanding of the grading of CCRCC and PRCC that can inform future research.

## Evolution of WHO/ISUP grading system

### Historical Composite Gradings

From 1949 and the ensuing decades, several 3‐ and 4‐tiered grading schemes for RCC were introduced beginning with the grading by Griffiths and Thackray^11^ and several others.^3^, ^12^, ^13^, ^14^, ^15^, ^16^, ^17^, ^18^, ^19^ Most of these historical grading systems assessed varying combinations of histological features that included tumour architecture, nuclear features, mitosis, cell type or necrosis, among others. These grading approaches showed differences in survival outcomes for RCC. However, these grading systems were applied then without the appreciation of the current RCC subtypes, and some of these schemes had vague criteria that could be challenging to use in a reproducible manner.

### Fuhrman Nuclear Grade

In 1968, Meyers *et al*.^20^ introduced one of the first of many several subsequent grading approaches that focused on nuclear features.^21^, ^22^, ^23^, ^24^, ^25^, ^26^, ^27^, ^28^, ^29^ Among these nuclear‐based gradings, it was the 4‐tiered grading by Fuhrman *et al*.^24^ in 1982 that gained wide recognition. In the original Fuhrman's study, RCCs were categorized into Fuhrman nuclear grades 1 (14%), 2 (50%), 3 (26%) and 4 (10%), with grading based on the most malignant or highest grade present, even if only focal. Metastatic rates were significantly different between Fuhrman nuclear grade 1 and combined Fuhrman nuclear grade 2–4 tumours, and 5‐year survival curves were statistically different but only when Fuhrman nuclear grade 2 and 3 tumours were combined as one group. Interestingly, in an interobserver agreement study, the nuclear grading by Syrjänen and Hjelt^22^ outperformed Fuhrman nuclear grade, the nuclear grading by Skinner *et al*.,^21^ and the grading based on tumour architecture and cell type by Arner *et al*.^14^ The Fuhrman nuclear grade suffered from the complexities of simultaneously assessing three different nuclear variables (size, shape and nucleolar prominence) that are not always concordant in their gradations. A reason that Fuhrman nuclear grade, as a composite of these three nuclear features, cannot be strictly applied in a subset of CCRCC,^30^ and likely contributed to the fair to moderate interobserver agreements when applying this system.

### WHO/ISUP Grade

Around the 2000s, the approach to grading was being simplified in practice to focus mainly on nucleolar prominence for grades 1–3. While nucleolar‐based grading was already being used by some expert pathologists then to derive Fuhrman nuclear grade, its use as a grading system distinct from Fuhrman nuclear grade was codified through two studies from the same group.^31^, ^32^ In 2006, the study by Sika‐Paotonu *et al*.^31^ on PRCC showed that focal nucleolar grade was independently associated with survival, unlike the other nuclear measures of Fuhrman nuclear grade in size and shape. In 2011, the study by Delahunt *et al*.^32^ for nucleolar grades 1–3 for CCRCC showed that the worst nucleolar grade discriminated in survival among grades 2 and 3 tumours. This nucleolar‐based grading for CCRCC and PRCC was subsequently adopted in the 2012 ISUP consensus conference as the ISUP grade,^6^ and with slight modification (nucleolar eosinophilia), it was incorporated into the 2016 WHO blue book as the WHO/ISUP grade^5^ and later retained in the 2022 WHO iteration (Table 1).^10^ It is recommended that the WHO/ISUP grade should be assigned within a single high‐power field showing the greatest degree of nuclear changes. The WHO/ISUP grading system has also been recognized to be inapplicable for chromophobe RCC (ChRCC).^6^ Currently, different alternative (2‐, 3‐ and 4‐tiered) grading approaches are being recommended for ChRCC.^4^ A decade into its WHO adoption for CCRCC and PRCC, studies on WHO/ISUP grade are still unfolding.

**Table 1:** WHO/ISUP grading system for clear cell RCC and papillary RCC

Grade	Description
Grade 1	Nucleoli absent or inconspicuous and basophilic at ×400 magnification
Grade 2	Eosinophilic nucleoli observed at ×400 but not prominent at ×100 magnification
Grade 3	Nucleoli prominent and eosinophilic at ×100 magnification
Grade 4	Carcinoma cells display extreme nuclear pleomorphism, tumour giant cells, and/or any amount of sarcomatoid or rhabdoid differentiation

## Applications of WHO/ISUP grading system

### Clear Cell RCC

After its introduction, several studies were conducted on WHO/ISUP grading for CCRCC. Dagher *et al*.^30^ showed a difference in cancer‐free survival (CFS) for grades 1–4 in 376 CCRCCs at mean follow‐up of 35 months that was significant on multivariate analysis. Delahunt *et al*.,^33^ on a cohort of 3,017 CCRCCs from the Mayo Clinic Registry, showed differences in cancer‐specific survival (CSS) across all grades on 10‐year follow‐up by regression models. Leibovich *et al*.,^7^ also on a large cohort of CCRCCs from the Mayo Clinic Registry, showed a significant association of WHO/ISUP grade categories with progression or death from RCC in multivariate analysis, and updated the Leibovich prognostic model for CCRCC to include WHO/ISUP grade. Other studies showed a significant association to outcome when grades 1/2 and 3/4 were combined in multivariate analysis.^34^, ^35^ However, the study by Khor *et al*.^36^ that preceded the WHO criteria and used the ISUP grade (without accounting for nucleolar eosinophilia) on 842 consecutive CCRCCs showed a significant association only for grade 4, and not for grade 3 versus grades 1/2 combined, to overall survival (OS). Overall, most studies confirmed the prognostic value of WHO/ISUP grade for CCRCC.

### Papillary RCC

Since the adoption of the WHO/ISUP grade, several grading studies using this system for PRCC were also conducted. The aforementioned study by Leibovich *et al*.^7^ from Mayo Clinic also graded a cohort of 607 PRCCs and showed significant association with progression or death from RCC with 3‐tier WHO/ISUP grade (1/2, 3 and 4) in multivariate analysis. The Leibovich prognostic model for PRCC was also updated to incorporate the WHO/ISUP grade. Yang *et al*.^37^ in a study of 185 PRCCs showed high WHO/ISUP grade to be associated with disease‐free survival (DFS) and OS in multivariate analysis. The study by Le *et al*.^38^ on 52 consecutive PRCCs showed WHO/ISUP grade (grades 1/2 versus 3/4) to be associated with progression‐free survival (PFS) in univariate analysis. A study by Cornejo *et al*.^39^ that preceded the 2016 WHO criteria and used the ISUP grade (not accounting for eosinophilia) on 154 PRCCs showed ISUP grade to be a significant predictor of CSS and OS in multivariate analysis. However, three studies with 176, 254 and 319 patients with PRCC failed to show the prognostic value of WHO/ISUP grading.^40^, ^41^, ^42^ Interestingly, the study by Murugan *et al*.^43^ on 199 PRCCs with any ‘type 1’ features showed that the highest WHO/ISUP grade (recommended approach) was not associated with outcome, whereas the predominant WHO/ISUP grade was associated with PFS and CSS in univariate analysis. Studies using WHO/ISUP grade on PRCC showed mixed results, but with more data supporting continued use of this grading system.

## Issues in WHO/ISUP grading of clear cell RCC

### Issues in WHO/ISUP Grade Elements

#### Grade heterogeneity

**Figure 1:**
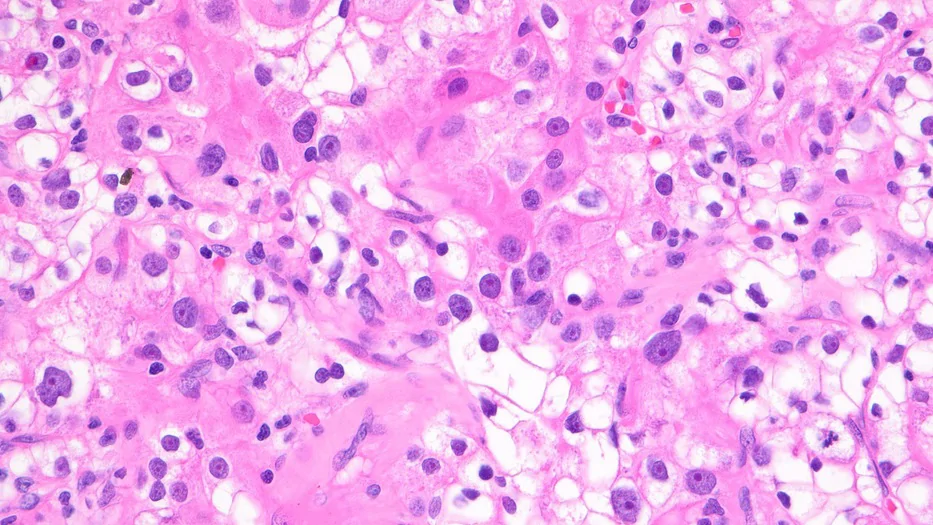
CCRCC with heterogeneous grade. Note association of eosinophilic cells with higher grade nuclei. [Colour figure can be viewed at wileyonlinelibrary.com]

Intratumoral heterogeneity in grades, patterns, genetics and biomarker expressions is not uncommon in CCRCC.^44^, ^45^, ^46^, ^47^, ^48^, ^49^, ^50^, ^51^, ^52^, ^53^ Eosinophilic cell area is known to harbour higher grade nuclei (Figure 1).^44^ Tumour patterns such as alveolar, pseudopapillary, insular or solid sheet‐like areas are associated with higher nuclear grade.^47^ Mutations in *BAP1*, *PTEN* or *SETD2* and higher tumour mutational burden when heterogeneously present are more likely to be detected in higher grade areas.^49^, ^50^, ^53^ The range of variations in grade within a tumour can be marked; for example, sarcomatoid change (grade 4) can be seen with background of grade 2.^54^ Grade heterogeneity is common even in small (<4 cm) clear cell RCC, and the highest grade is not the predominant grade in 37.5% of samples.^45^ Interestingly, Wu *et al*.^46^ observed discrepancies between WHO/ISUP grades at primary tumour and at venous tumour thrombus in 47% of CCRCCs and showed the discrepancy to be an independent risk factor in predicting outcome. Because of the heterogeneity in grades, it is recommended that grading should be based on the highest‐grade present.^6^, ^10^ However, the optimal minimum area for the highest grade remains to be established. In the 2012 ISUP conference, no consensus was reached regarding the minimum area for grading.^6^ Currently, it is recommended that the highest grade should be present in at least one high power field, which is approximated to be about 0.23 mm^2^ in most modern microscopes.^6^, ^10^ To address the issue of heterogeneity in CCRCC, some authors proposed modified extensive tumour sampling techniques.^55^, ^56^ At this point, it is important to ensure adequate tumour sampling (traditionally at least 1 section per cm of tumour greatest dimension and to make sure that all macroscopically distinct areas are sampled).

#### Grades 1–3 components

Some studies combined WHO/ISUP grades 1 and 2. Given that the number of grade 1 tumours can be small, this begs the question of whether these two lower grades should be merged (grades 1/2, 3 and 4).^34^, ^35^ The current AUA risk stratification for RCC does not make a distinction between grades 1 and 2,^2^, ^57^ in contrast to the Leibovich score for CCRCC.^7^ However, studies have shown a significant difference in outcome between WHO/ISUP grades 1 and 2, and continued separation and use of these two lower grades should be made including for risk prognostication for CCRCC.^7^, ^30^, ^33^ The high prevalence of grade heterogeneity also raises the question if extent or percent (%) of grade 3 has any prognostic value. One study showed that dividing WHO/ISUP grade 3 into <10%, 10%–50% and >50% or ≤50 or >50% in CCRCC is not associated with outcome.^58^

#### Grade 4 components

##### Extent (%) of grade 4

Two studies assessed % of high‐grade component in CCRCC. Dagher *et al*.^58^ divided WHO/ISUP grade 4 component into <10%, 10%–50% and >50% in 62 grade 4 CCRCCs and showed a significant difference in survival between <10% and >50% grade 4 in multivariate analysis. Whereas in the same study, the difference in survival was not demonstrated with % grade 3. Interestingly, no difference in outcome was shown between grade 3 and <10% grade 4 CCRCCs. Serrano *et al*.^59^ instead combined Fuhrman nuclear grades 3 and 4 and divided % Fuhrman nuclear grade 3/4 into 1%–50% and 51%–100% and by Cox proportional hazard multivariate analysis showed that % Fuhrman nuclear grade 3/4 is significantly associated with metastasis‐free survival (MFS), CSS and OS. Limited data suggest that % grade 4 in CCRCC has prognostic value; however, more data are needed to validate this finding.

##### Large multinucleated cells

**Figure 2:**
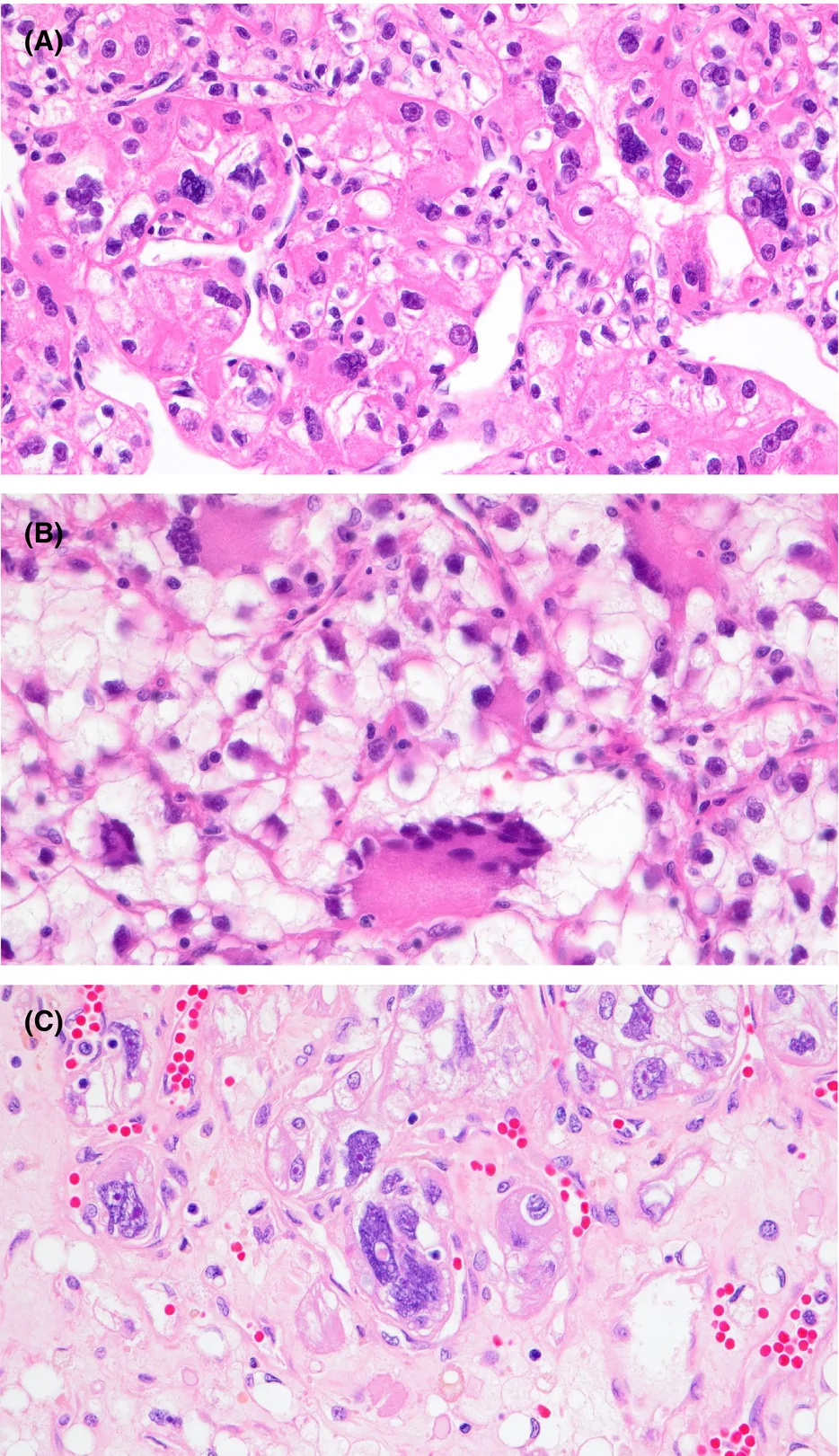
(**A**) Large multinucleated cells with degenerative atypia, (**B**) Syncytial‐type multinucleated cells and (**C**) multinucleated cells with extreme pleomorphism or bizarre‐appearing nuclei.

The current criteria for WHO/ISUP grade 4 includes ‘tumour giant cells’ that encompass multinucleated large cells with extreme pleomorphism or bizarre nuclei.^10^ However, there are other types of large multinucleated cells that can be seen in CCRCC, namely, syncytial‐type multinucleated cell (STMC) and multinucleated cell with degenerative atypia (MCDA) (Figure 2).^60^, ^61^, ^62^, ^63^ Williamson *et al*.^60^ first reported the uncommon STMC, which can be seen in 1.2%–1.5% of CCRCCs^62^ STMCs usually have voluminous eosinophilic granular cytoplasm and have nuclei and immunophenotype similar to the background mononuclear clear cells. The multiple nuclei can be arranged peripherally, and emperipolesis of leukocytes is a common feature. A subsequent study by Nova‐Camacho and Sangoi^61^ reported that STMCs, when present, can be seen in up to 20% of tumour area and are mainly seen around areas of necrosis. Both studies highlighted the association of STMCs with a subset of aggressive CCRCCs. In the architectural grading study by Verine *et al*.,^64^ STMCs were identified in higher proportion (15%) of CCRCCs, and based on likelihood for DFS, STMC is considered as intermediate grade (grade 2 of 3).

Pacheco *et al*.^62^ described the sporadic presence of MCDA in 28% of CCRCCs. These large cells are characterized by multiple tightly clustered and overlapping unenlarged nuclei with dense clumped chromatin. The authors also described multinucleated tumour cells with no anaplasia, although these cells are likely to be CCRCC cells that are bi‐ or trinucleated distinct from the other multinucleated cells. Tumours with MCDA did not show increased risk of recurrence or metastasis, unlike for multinucleated cells showing anaplasia, and the authors concluded that MCDA is not relevant for grading. Cai *et al*.^47^ showed that the presence of STMCs had worse DFS compared to MCDA in CCRCCs and that tumours with MCDA had less favourable DFS compared to RCCs without both multinucleated cells.

While the classic multinucleated cells with extreme pleomorphism should be graded as WHO/ISUP grade 4, MCDA should not be considered as grade 4. Grading of STMC is controversial and these cells are possibly not grade 4. A differential diagnosis for multinucleated tumour cells is the megakaryocytes that can rarely occur in CCRCC with extramedullary haematopoiesis.^65^

##### Sarcomatoid change

###### Grade spectrum of spindle cells

Sarcomatoid change is long recognized to be associated with poor outcome in RCC and shows specific genomic and epigenomic alterations, notably in the Hippo pathway.^54^, ^66^, ^67^, ^68^, ^69^, ^70^ Recent developments revealed that immune checkpoint inhibitors provide survival and response benefits in sarcomatoid RCC.^71^, ^72^ Sarcomatoid change is usually composed of spindle cells that resemble undifferentiated sarcoma, and rarely may contain malignant heterologous elements such as rhabdomyosarcoma or osteosarcoma.^54^, ^66^ It is unclear whether the presence of heterologous elements has an impact on outcome among sarcomatoid RCCs. One study showed no association of malignant heterologous elements with death from RCC among sarcomatoid RCCs, but with a limited number of cases.^66^

**Figure 3:**
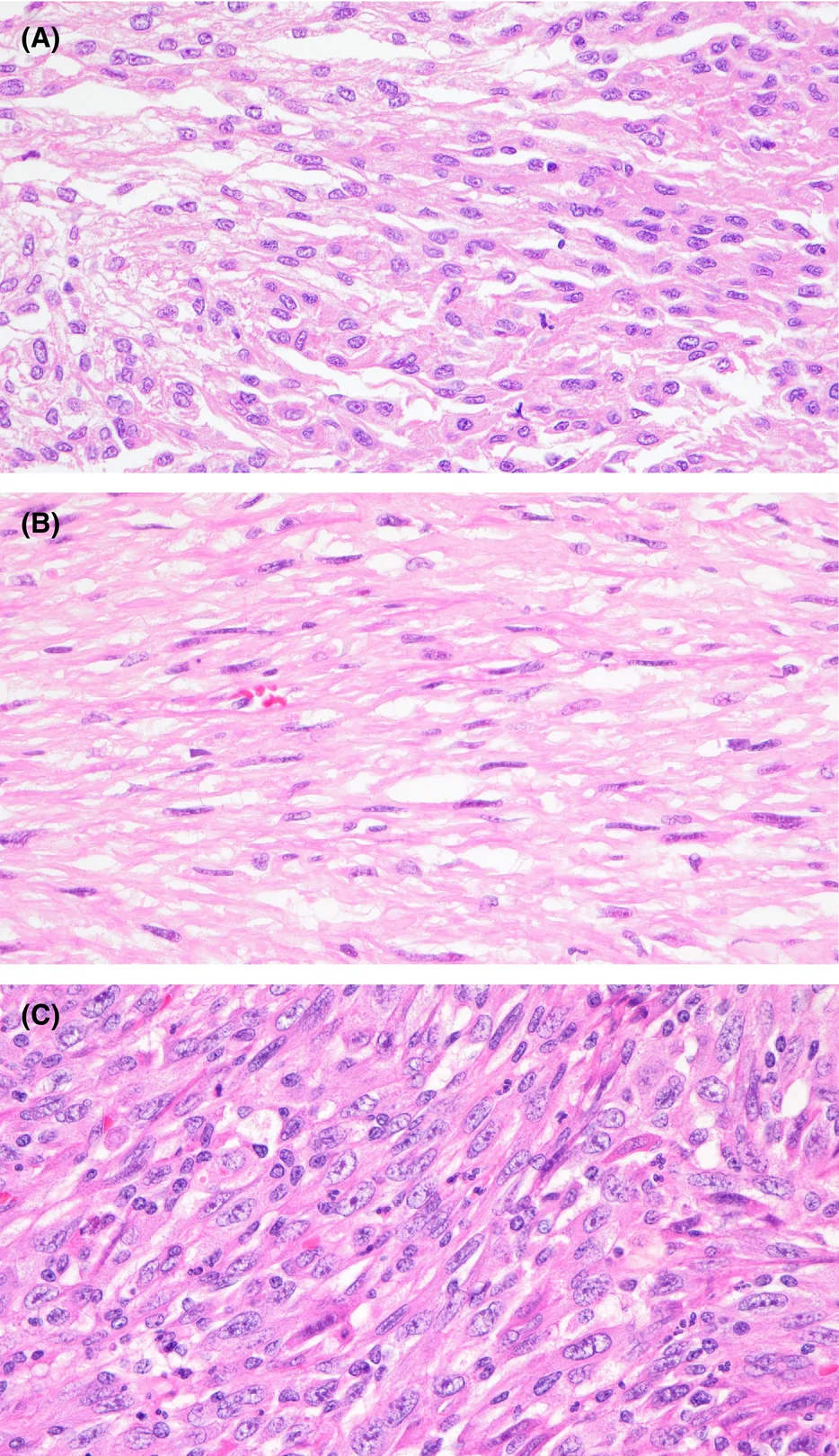
(**A**) Elongated or spindle cells intermingled with epithelioid cells and (**B**) spindle cells with low‐grade appearing nuclei. (**C**) Typical sarcomatoid spindle cells with pleomorphic nuclei.

The spindle cells in sarcomatoid change typically have higher grade nuclei, but uncommonly may have low‐grade‐appearing nuclei (Figure 3). Tanas Isikci *et al*.^73^ described a series of ‘low‐grade’ spindle cell proliferation in CCRCC with comparable immunohistochemical and molecular profile to conventional CCRCC cells, including with epithelial–mesenchymal transition (EMT) markers, and concluded that these cells may not represent an initial phase of sarcomatoid change. Cai *et al*.^47^ described ‘spindle cell change’ distinct from sarcomatoid change in 3.5% of CCRCCs that had better DFS than sarcomatoid change. However, other studies suggest a more aggressive behaviour for ‘low‐grade spindle cells’. Cheville *et al*.^66^ divided sarcomatoid change in 102 sarcomatoid RCCs that were predominantly CCRCC into low grade (13.7%) and high grade (86.3%), and grade did not show correlation with death from RCC. Sirohi *et al*.^74^ based on prediction of OS, categorized both ‘spindled low‐grade’ and sarcomatoid change to be unfavourable histological growth patterns.

Spindle cells with low‐grade nuclei can occur in CCRCC, and additional studies are needed to elucidate on its prognostic significance. While grading should be based on nuclear/nucleolar features, there is uncertainty on its application to these low‐grade appearing spindle cells because of limited data. Further, the issue of observer reproducibility must be addressed to avoid undegrading bona fide sarcomatoid change with poor prognostic effect.

###### Sarcomatoid versus non‐sarcomatoid grade 4

Sarcomatoid change is seen in about 35%–60% of grade 4 CCRCC.^66^, ^75^, ^76^, ^77^ Studies have shown a worse outcome of sarcomatoid grade 4 RCC compared to non‐sarcomatoid grade 4 RCC.^66^, ^75^, ^76^, ^77^ Cheville *et al*.^66^ reported in 174 patients with grade 4 CCRCCs that the presence of a sarcomatoid component (59.8%) was independently associated with outcome; CSS at 2 years post‐nephrectomy was 32.9% for sarcomatoid RCC and 42.2% for non‐sarcomatoid grade 4 RCC. Zhang *et al*.^75^ later showed in 411 patients with ISUP grade 4 RCC (also from the same institution) that the presence of sarcomatoid change (49% of tumours) was associated with a 58% increased risk of death from RCC. The increased risk of death was higher at 82% for non‐metastatic grade 4 RCCs. Kara *et al*.^77^ showed that the presence of sarcomatoid change in 246 patients with Fuhrman nuclear grade 4 RCCs was associated with worse median CSS in both metastatic and non‐metastatic cases. Recently, the study by Blum *et al*.^76^ on 564 non‐metastatic WHO/ISUP grade 4 RCCs demonstrated that at every stage, sarcomatoid RCC had worse CSS compared to non‐sarcomatoid grade 4 RCC. Notably, localized pT1 sarcomatoid RCC had worse CSS than pT3 non‐sarcomatoid grade 4 RCC.

There is convincing evidence that the presence of sarcomatoid change portends a worse outcome even for grade 4 RCCs. Whether sarcomatoid change warrants another higher‐grade distinction separate from grade 4 will need further consideration. Importantly, the presence of sarcomatoid change should be reported even in grade 4 RCCs, including in biopsy specimens, because of its negative effect on outcome and for immune checkpoint inhibitor treatment options. Both ICCR and CAP require reporting the presence of sarcomatoid change even for grade 4 RCC.^8^, ^9^

###### Extent (%) of sarcomatoid

The extent of sarcomatoid change in RCC can range from 1% to 100%.^54^ Several studies showed the prognostic value of dividing extent (%) of sarcomatoid component in RCC (Table 2).^66^, ^75^, ^78^, ^79^, ^80^, ^81^, ^82^, ^83^, ^84^ However, varying cut‐offs were used for % sarcomatoid including 10%, 20% and 30%. Zhang *et al*.^75^ showed that for every 10% increase in sarcomatoid component there is an associated 6% increase in risk of death. Patel *et al*.^81^ reported that sarcomatoid component was associated with worse OS per 10% increase and at ≥20% cut‐off. Adibi *et al*.^82^ showed that presence of >40% sarcomatoid component had the least likelihood to be alive at 1 year. However, some older studies reported that % sarcomatoid component had no effect on outcome.^66^ Shuch *et al*.^84^ divided sarcomatoid component into quartiles while Cheville *et al*.^66^ divided sarcomatoid component into 5%–10%, 15%–50% and >50% and both studies failed to show effect of % sarcomatoid component on outcome.

**Table 2:** Studies that divide the extent of sarcomatoid component in RCC

Study	Sarcomatoid RCC (% clear cell RCC)	Cut‐off	End point	Significant	MVA	Comment
Lucarelli *et al*.^77^	212 (100%)	20%	CSS, RFS	Yes	Yes	
Soytas *et al*.^78^	201 (80%)	10%	Metastasis, death	Yes	No	No association using 5% or 15% cut‐offs
Salgia *et al*.^79^	104 (predominant)	10%	OS, MFS (organ‐confined tumours)	Yes	Yes	
Patel *et al*.^80^	128 (78%)	20%	OS	Yes	Yes	Significant for cM0 and cM1
Adibi *et al*.^81^	186 (73%)	10%	OS	Yes	Yes	>40% least likely to be alive at 1 year
Kim *et al*.^82^	59 (74%)	25%	OS	Yes	Yes	In cM0 only and not with cM1. No difference observed using 5% or 10% cut‐offs
Zhang *et al*.^74^	215 (79%)	30%	CSS	Yes	Yes	10% increase sarcomatoid associated with 6% increased risk of death
Shuch *et al*.^83^	104 (65%)	<25%, 25%–<50%, 50%–<75%, ≥75%	Survival	No	Yes	Sarcomatoid divided into quartile
Cheville *et al*.^65^	120 (86%)	5%–10%, 15–50%, >50%	Death from RCC	No	No	

Abbreviations: CSS, cancer‐specific survival; MFS, metastasis‐free survival; MVA, multivariate analysis; OS, overall survival; RFS, recurrence‐free survival.

There is enough evidence to show that % sarcomatoid has an impact on prognosis of RCC, and thus, this element should be reported. However, the optimal cut‐off or divisions (10%, 20% or 30%) for % sarcomatoid has yet to be established. Of note, a recent phase II clinical trial combining immune checkpoint inhibitor atezolizumab and the anti‐VEGF bevacizumab showed objective response in RCC with ≥20% sarcomatoid component.^71^ Both ICCR and CAP recommend reporting % sarcomatoid component in nephrectomy specimens as an optional element, and without requiring any cut‐offs.^8^, ^9^ Reporting % sarcomatoid component is not recommended when present in biopsy specimens.

##### Rhabdoid feature

**Figure 4:**
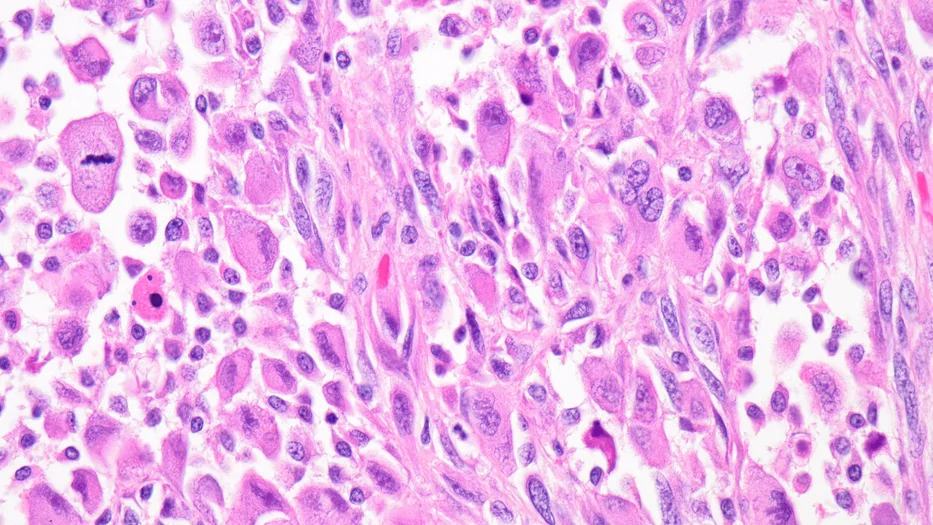
Rhabdoid cells admixed with sarcomatoid spindle cells. [Colour figure can be viewed at wileyonlinelibrary.com]

Rhabdoid change in RCC is recognized as a poor prognostic factor and is associated with alterations in SWI/SNF chromatin remodelling complex.^85^, ^86^ Rhabdoid change is characterized by large epithelioid cells with vesicular nuclei, prominent nucleoli and large paranuclear intracytoplasmic inclusions (Figure 4).^85^ In grade 4 RCCs, rhabdoid change (only) is reported in 20–28%, and rhabdoid in combination with sarcomatoid change in 7%–16%.^77^, ^87^ When present together, rhabdoid change is usually contiguous with sarcomatoid change.

Both rhabdoid and sarcomatoid change are graded as WHO/ISUP grade 4 and begs the question whether these two patterns are comparable in terms of effects on outcome. Zhang *et al*.^87^ showed that rhabdoid change in ISUP grade 4 RCCs was not associated with death from RCC, in contrast to sarcomatoid change. However, patients with RCC with rhabdoid change are significantly more likely to die from RCC than patients with grade 3 RCC. Kara *et al*.^77^ also showed that rhabdoid change was not associated with additional mortality in grade 4 RCCs, unlike sarcomatoid change. Verine *et al*.^64^ showed a lower likelihood for DFS for rhabdoid change compared to sarcomatoid change and categorized these two patterns into different grade categories in their architectural grading.

Studies support the WHO/ISUP grade 4 designation of rhabdoid change, and data show that rhabdoid change does not convey an identical negative effect to outcome with sarcomatoid change. Therefore, these two high‐grade patterns should not be combined when reporting. There is no data to support reporting extent (%) of rhabdoid change in RCC.

### Issues in WHO/ISUP Grade Applications

#### Observer agreement in grading

Since WHO/ISUP grade is relatively recent, most prior interobserver studies were performed with Fuhrman nuclear grade, and agreements on 4‐tier Fuhrman nuclear grade were fair to moderate (*k* = 0.22–0.44).^88^, ^89^, ^90^, ^91^ A study by three uropathologists using WHO/ISUP grade for CCRCC showed substantial agreement (*κ* = 0.62) on digital slides and moderate agreement (*κ* = 0.50) on glass slides.^92^ For the intraobserver study, kappa agreement for pathologists A, B and C between two glass slides was 0.69, 0.69 and 0.81, respectively. In two separate studies by three pathologists, both studies showed substantial and almost perfect agreements (*κ* = 0.65 and *κ* = 0.87–0.90) on WHO/ISUP grade for CCRCC.^64^, ^93^ Since the assessment of WHO/ISUP grades 1–3 relies on a single variable, improvement in agreement is expected, and this is supported by the few studies available.

#### Accuracy of needle biopsy grade

Most studies comparing grades in NB and nephrectomy encompassed the prior Fuhrman nuclear grade. One meta‐analysis showed fair agreement (*κ* = 0.34) with concordance rate of 62.5% for 4‐tier grade between NB and nephrectomy.^94^ One systematic review reported concordance of 51.5%–75.9% between NB and nephrectomy grades.^95^ Proye *et al*.^96^ reported accuracy of 52.6% for 4‐tier ISUP grade in >1400 NBs, and accuracy increased to 67.9% when ISUP grades were dichotomized to low (1/2) and high (3/4) grades. Another study from a large centre showed NB accuracy for grade was 94% for low and high grades grouping.^97^ There is significant discordance between grades in NB and nephrectomy for RCC. Most discordant grades were due to under grading (92% in one study) and very likely attributed to intratumoral heterogeneity in grade discussed above.^97^, ^98^ The ICCR considers grade as provisional for reporting in biopsy because it can change in the nephrectomy specimen due to heterogeneity.^8^ Reporting a provisional grade is of value, especially if it is of higher grade since correlation with nephrectomy grade is better, and can be helpful in guiding subsequent management.

#### Grading of multifocal tumours

The grades among multifocal synchronous RCCs can vary. In one study of 50 patients with multiple sporadic synchronous renal masses, grades among the tumours were concordant in only 62.5% of cases.^99^ Another study on 128 patients with sporadic multifocal tumours showed that the largest (index) tumour had the more aggressive histology in 77.3% of cases.^100^ In another study on 97 patients, this time with sporadic unilateral multifocal renal tumours (median 3 tumours), grade among the tumours was concordant (low or high) in only 51.5% of cases.^101^ Multifocal RCCs can vary in grade and the largest tumour may not always have the highest grade. There is no official recommendation on how to report grades in cases of multifocal RCCs, especially with discordant grades (highest grade is in smaller RCC), whether to report the highest grade only or enumerate the different grades. A potential issue with reporting only the highest grade (in smaller RCC) is it may misrepresent the largest or higher stage RCC (index tumour), and its effect on risk stratifications if any, where both grade and stage are used, is still unclear. Currently, both ICCR dataset and CAP protocol allow reporting of a single WHO/ISUP grade in a nephrectomy case regardless of tumour multifocality.^8^, ^9^

## Issues in WHO/ISUP grading of papillary RCC

### Grading Issues in Common with Clear Cell RCC

There is now enough evidence to support the abandonment of subtyping PRCCs into types 1 and 2, a topic that is already well‐covered in the literature and will not be discussed here.^37^, ^102^, ^103^, ^104^ Some of the issues on grading described above in CCRCC can also occur in PRCC, such as issues on grade heterogeneity, observer reproducibility and biopsy undergrading, although comparatively with lesser data. Ball *et al*.^45^ reported that heterogeneity in Fuhrman nuclear grade in small (<4 cm) PRCC is common, and in 25% of samples the higher grade is not the predominant (<50%) grade. A review by six urologic pathologists on 30 papillary RCCs for nucleolar grade (grades 2 vs 3) showed only slight interobserver agreement (*κ* = 0.16).^105^ Prendeville *et al*.^106^ reported grade concordance of 64% for four pathologists between NB and nephrectomy for 4‐tier WHO/ISUP grades in PRCC. Upgrading of NB grade in nephrectomy occurred in 12% with no cases of downgrading, a scenario similar in NB for CCRCC.

### Grade in Small (≤1.5 cm) Papillary Tumours

PRCC and papillary adenoma overlap morphologically, immunohistochemically and cytogenetically. Low‐grade nucleus (1 or 2) is one criterion to distinguish papillary adenoma from PRCC in addition to size (≤1.5 cm) and absence of a capsule.^10^ This begs the question of whether small papillary epithelial tumours with WHO/ISUP grade 3 nuclei should warrant a diagnosis of PRCC. No studies had directly addressed this issue; however, two large studies from the Swedish National Kidney Cancer Registry and multi‐institutional data of United States and European centres showed 4% and <1% metastasis in renal tumours of up to 2 cm in size.^107^, ^108^ Fuhrman nuclear grade 3 (15.9%) and 4 (0.5%) have been reported in RCCs <2 cm in size.^108^ These studies were contradicted by data from MSKCC and Mayo clinic of >500 patients that showed no metastasis occurring in <2 cm renal tumours.^109^, ^110^ Because of the small incidence of malignant capability in <2 cm renal tumours, low‐grade nuclei should be required to diagnose papillary adenoma, and currently, PRCC may be considered for <2 cm tumours with higher grade nuclei until data become available.

## Modified grading incorporating necrosis

**Figure 5:**
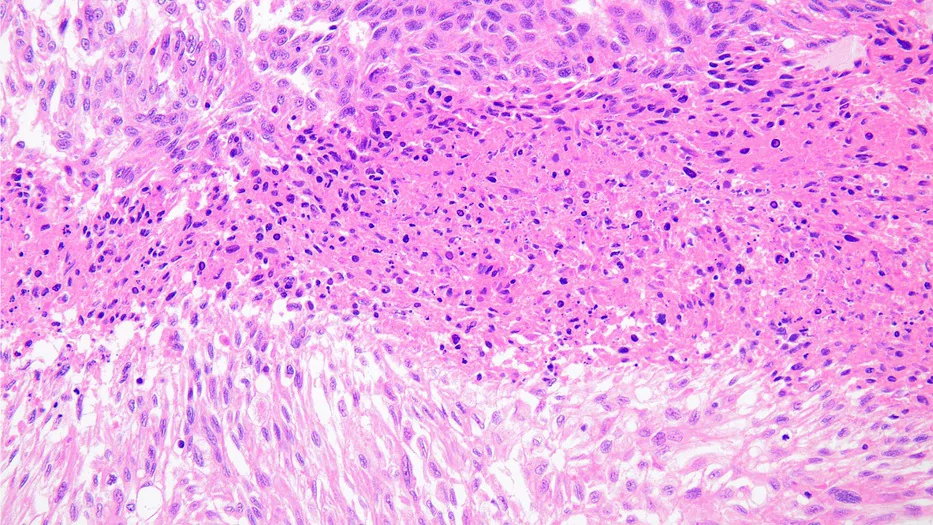
Tumour‐associated necrosis (granular necrosis) showing loss of architecture with cytoplasmic and nuclear debris. [Colour figure can be viewed at wileyonlinelibrary.com]

Tumour necrosis is frequent in RCC and is of prognostic significance in CCRCC.^6^, ^70^, ^111^ Tumour‐associated necrosis is typically well demarcated and shows loss of architecture with granular nuclear and cytoplasmic debris (granular necrosis) distinct from coagulative necrosis (Figure 5).^112^ Delahunt *et al*.^33^ proposed a 4‐tiered modified grading system for clear cell RCC wherein necrosis is incorporated into ISUP grade (Table 3). A significant difference in CSS between each of the modified grades was demonstrated in the study. The proposed modified grading also had a superior concordance index and performed better than ISUP grade.

**Table 3:** Modified grading system integrating necrosis for clear cell RCC

Grade	Description
Grade 1	ISUP grade 1 + ISUP grade 2 without necrosis
Grade 2	ISUP grade 2 with necrosis + ISUP grade 3 without necrosis
Grade 3	ISUP grade 3 with necrosis + ISUP grade 4 without necrosis
Grade 4	ISUP grade 4 with necrosis or sarcomatoid/rhabdoid tumours

A subsequent study by Dagher *et al*.^111^ on 376 CCRCCs showed no significant difference in outcome between WHO/ISUP grade 2 with necrosis and grade 3 without necrosis (modified grade 2 components), and between WHO/ISUP grade 3 with necrosis and grade 4 without necrosis (modified grade 3 components). The study by Khor *et al*.^36^ on 842 CCRCCs using the modified grading showed statistically significant differences in survival between patients with modified grades 1/2, 3 and 4. The authors further identified three statistically significant prognostic groups: (1) non‐necrotic ISUP grade 1–3, (2) ISUP grade 1–3 with necrosis and ISUP grade 4 with <10% necrosis, and (3) ISUP grade 4 with necrosis or sarcomatoid/rhabdoid change. Sirohi *et al*.^74^ showed better separation in Kaplan–Meier analysis for modified grade and stronger statistical significance than WHO/ISUP grade in Cox proportional hazard model. Verine *et al*.^64^ showed comparable Harell concordance index on 217 localized CCRCCs for WHO/ISUP grade and modified grade for DFS and CSS.

By incorporating necrosis, the prognostic ability of WHO/ISUP grade is enhanced. Modified grading is promising; however, additional studies on this approach are needed including on its integration into risks models. Currently, both ICCR dataset and CAP protocol require reporting of necrosis as a separate element.^8^, ^9^

## Architectural or pattern‐based grading and risk assessment

Pattern‐based approach in grading is not a new concept and has been used previously in historical gradings for RCCs.^11^, ^12^, ^13^, ^14^, ^15^, ^16^, ^17^, ^18^, ^19^ Recently, there is resurgence in pattern‐based schemes for grading and prognostication of CCRCCs (Table 4).^47^, ^64^, ^74^, ^93^ Verine *et al*.^64^ proposed a 3‐tiered architecture‐based scoring that outperformed WHO/ISUP grade and modified WHO/ISUP grade + necrosis and independently predicted DFS and CSS. Interobserver agreement for the 3‐tiered architectural score is better than the 3‐tiered WHO/ISUP grade (*κ* = 0.82 vs. *κ* = 0.71). Cai *et al*.^47^ identified among 9 architectural and 24 cytological and tumour microenvironment patterns in CCRCC that tubular architecture, necrosis, renal parenchymal infiltration and chromophobe‐like feature are independent unique predictors of DFS when nucleolar grade was considered. From these variables, a predicted nomogram for CCRCC patients after nephrectomy was created. From combinations of 24 high‐grade patterns in CCRCC, Sirohi *et al*.^74^ created 3‐tiered and 2‐tiered risk models that performed equally well as modified WHO/ISUP grade + necrosis and better than WHO/ISUP grade in predicting OS. From nine architectural patterns, Ohe *et al*.^93^ created a 3‐category vascular‐based architectural classification that showed the best accuracy in predicting RFS and was integrated into a risk model with greater predictive accuracy than conventional risk models. This vascularity‐based architectural classification showed good agreement among 3 pathologists (*κ* 0.83–0.93 for vascularity‐based score and *κ* 0.87–0.90 for WHO score). Others also showed that renal parenchymal infiltration or micronodular spread are associated with poorer outcome in clear cell RCC.^113^, ^114^

**Table 4:** Pattern‐based grading and prognostication for clear cell RCC

Study	Growth patterns
Verine *et al*.^63^	**Grade 1** *+: macrocystic, microcystic, tubulocystic, compact, acinar, clear cell papillary RCC‐like, regressive
	**Grade 2** *+: large nest, alveolar or micropapillary, chromophobe/oncocytic cell‐like, intracytoplasmic hyaline globule, intratumoral inflammatory reaction
	**Grade 3** *: rhabdoid, multinucleate giant cells, enlarge vascular space
	**Grade 4** *: sarcomatoid, infiltrative growth with entrapped glomeruli, LVI
Cai *et al*.^46^	**Aggressive** **: alveolar, papillary, insular, solid, tumour giant cells, ChRCC‐like, HLRCC‐like, tRCC‐like, hyaline globules, intratumoral lymphoplasmacytic, neutrophilic infiltrate, nodular growth, LVI, tumour infiltration into parenchyma
	**Favourable** **: microcystic, tubular, bleeding follicles, compact nest
	**Predictive nomogram**: tubular pattern, necrosis, renal parenchymal infiltrative edge, ChRCC‐like, grade
Sirohi *et al*.^73^	**Favourable** ***: small nests, expansile nests, nests with high nuclear to cytoplasmic ratio
	**Intermediate** ***: oncocytic papillary, Paneth cell like features, solid pseudopapillary, oncocytic, alveolar, regressive, tubulopapillary, calcification, papillary, cystic, glandular, hemangiomatous, endothelial proliferation, tubular, giant cells
	**Unfavourable** ***: infiltrative, spindled low‐grade, sarcomatoid, fused nests/sheets, rhabdoid, necrosis
	**2‐tiered risk model**: high risk if WHO/ISUP grade 4, necrosis or any unfavourable pattern; all others are low risk
	**3‐tiered risk model**: high risk if with any unfavourable pattern, low risk if does not qualify as high risk and present with any favourable pattern and have up to 5 patterns including no more than 2 indeterminant patterns; all others at moderate risk
Ohe *et al*.^92^	**Category 1** ****: compact/small nested, microcyst/microcyst, tubular/acinar
	**Category 2** ****: alveolar/large nested, thick trabecular
	**Category 3** ****: solid sheets, rhabdoid, sarcomatoid

* According to likelihood ratios for DFS; + Grade 1 and 2 combined for preferred 3‐tiered grading system.

** By univariate analysis for DFS.

*** By HR predictive of OS.

**** By vascular network score with CD31 immunohistochemistry.

Several unfavourable architectures were recently reported for PRCC. Yang *et al*.^37^ identified solid, micropapillary or hobnail architecture in PRCC to be associated with worse DFS and OS. Chan *et al*.^105^ showed microcysts, hobnail, solid and micropapillary in PRCC as significantly associated with adverse outcomes, but microcysts are more common and have superior interobserver reproducibility. Murugan *et al*.^43^ reported that sheet‐like architecture in PRCC was significantly associated with CSS.

Preliminary studies suggest that architectural or pattern‐based grading provides useful prognostic information that has the potential to be superior to the WHO/ISUP grading and modified grading that integrates necrosis. However, confirmation studies from other authors and possible standardization of these different schemes are needed. Further, the complexity in simultaneously assessing multiple histological and cellular variables for grading may become impractical for routine practice. An advantage of the current WHO/ISUP grade is that only a single variable (nucleoli) is assessed for grades 1–3, which provides practical advantage over Fuhrman nuclear grade and other historical composite gradings that also used architectural and other features.

## Conclusion

This review presents recent updates and issues in use of WHO/ISUP grading and novel approaches to histological grading and risk assessments for CCRCC and PRCC (Tables 5 and 6). Other innovative approaches not covered in this review include artificial intelligence or deep neural networks in grading RCC on histopathological slides and radiomics.^115^ This current review may serve as a best practice guide in addressing some of the grading issues and help identify potential gaps for research that will help optimize grading of CCRCC and PRCC.

**Table 5:** Summary of updates and issues in grading CCRCC

Issues	Comments and suggestions
*I. WHO/ISUP grading validation*	
1. Grading of CCRCC	Most studies confirmed use
2. Grading of PRCC	Some studies confirmed use but contradicted by others
3. Grading of ChRCC	WHO/ISUP grade not useful and novel 2‐, 3‐ and 4‐tiered grading approach are being recommended
*II. WHO/ISUP grade elements in CCRCC*	
1. Grade heterogeneity	Highest grade* is not always the predominant grade
2. Combining (lowest) grades 1 and 2	Differences in outcome shown between grades 1 and 2 and these grades should not be combined
3. % Grade 3	Limited data shows no prognostic value
4. % Grade 4	Limited data suggests prognostic value
5. Multinucleated cells	Multinucleated cells with pleomorphic nuclei graded as 4
	Syncytial‐type multinucleated cells are likely not grade 4
	Multinucleated cells with degenerative atypia should not be graded as 4
6. Spindle cells	Uncommon ‘low‐grade‐appearing’ spindle cells occur; behaviour not clear as grade 4
7. Sarcomatoid vs non‐sarcomatoid grade 4	Sarcomatoid has worse behaviour than non‐sarcomatoid grade 4
8. Extent (%) sarcomatoid	% sarcomatoid has prognostic value, but optimal % cut‐off (10%, 20% or 30%) is not yet identified
	20% cut‐off suggested for immune checkpoint inhibitor
9. Rhabdoid	Designate as grade 4 but behaviour is not similar with sarcomatoid
*III. WHO/ISUP grade applications*	
1. Interobserver agreement	Data so far shows better agreement than Fuhrman nuclear grade
2. Needle biopsy grade	Significant discordance with nephrectomy grade, likely due to grade heterogeneity
3. Multifocal tumours	Grades can be discordant and highest grade may occur in non‐index tumour
	No recommendation on reporting (report highest grade or all grades)

* In at least 1 HPF; optimal area not established.

**Table 6:** Summary of updates and issues in PRCC and new approaches to grading

Issues	Comments and suggestions
*I. WHO/ISUP grading of PRCC*	
1. Grade heterogeneity and biopsy	Likely similar undegrading in biopsy as in CCRCC but limited data
2. Small (≤1.5 cm) papillary tumours	Papillary adenoma requires low‐grade nuclei for diagnosis*
*II. Novel approaches*	
1. Modified grading incorporating necrosis	Enhanced prognostic ability than WHO/ISUP but additional studies needed
2. Pattern‐based grading and risk assessment	Shows potential to be better than WHO/ISUP for prognostication
	Needs confirmation and possible standardization
	More complex to use than WHO/ISUP grade

* Consider PRCC for higher grade nuclei.

## Author contributions

G.P.P.: conceptualization, data curation, formal analysis, writing – original draft and editing. M.B.A.: conceptualization, writing – review and editing. J.W.K.: writing – review and editing. L.C.: writing – review and editing. H.M.: conceptualization, writing – review and editing. All authors reviewed and approved the final version.

## Conflict of interest statement

The authors have no conflicts of interests to disclose.

## Consent

The manuscript content, figures and tables are all original and meet the journal's copyright requirements.

## Data Availability

Data sharing not applicable to this article as no datasets were generated or analysed during the current study.
